# Predicting Masaoka-Koga Clinical Stage of Thymic Epithelial Tumors Using Preoperative Spectral Computed Tomography Imaging

**DOI:** 10.3389/fonc.2021.631649

**Published:** 2021-03-25

**Authors:** Qing Zhou, Xiaoai Ke, Jiangwei Man, Bin Zhang, Furong Wang, Junlin Zhou

**Affiliations:** ^1^Lanzhou University Second Hospital, Lanzhou, China; ^2^Second Clinical School, Lanzhou University, Lanzhou, China; ^3^Key Laboratory of Medical Imaging of Gansu Province, Lanzhou, China

**Keywords:** thymic epithelial tumor, spectral CT, imaging, Masaoka-Koga clinical staging, predicts

## Abstract

**Objectives:**

To investigate the utility of spectral computed tomography (CT) parameters for the prediction of the preoperative Masaoka-Koga stage of thymic epithelial tumors (TETs).

**Materials and Methods:**

Fifty-four patients with TETs, aged from 37 to 73 years old, an average age of 55.56 ± 9.79 years, were included in the study.According to the Masaoka-Koga staging method, there were 19 cases of stage I, 15 cases of stage II, 8 cases of stage III, and 12 cases of stage IV disease. All patients underwent dual-phase enhanced energy spectral CT scans. Regions of interest (ROIs) were defined in sections of the lesion with homogeneous density, the thoracic aorta at the same level as the lesion, the outer fat layer of the lesion, and the anterior chest wall fat layer. The single-energy CT value at 40-140 keV, iodine concentration, and energy spectrum curve of all lesion and thoracic aorta were obtained. The energy spectrum CT parameters of the lesions, extracapsular fat of the lesions, and anterior chest wall fat in stage I and stage II were obtained. The energy spectrum CT parameters of the lesions, enlarged lymph nodes and intravascular emboli in the 3 groups were obtained. The slope of the energy spectrum curve and the normalized iodine concentration were calculated.

**Results:**

In stage I lesions, there was a statistically significant difference between the slope of the energy spectrum curve for the lesion and those of the fat outside the lesion and the anterior chest wall in the arteriovenous phase (P<0.001, P<0.001). The energy spectrum curve of the tumor parenchyma was the opposite of that of the extracapsular fat. In stage II lesions, there was a statistically significant difference between the slope of the energy spectrum curve for the anterior chest wall and those of the lesion and the fat outside the lesion in the arteriovenous phase(P<0.001, P<0.001). The energy spectrum curve of the tumor parenchyma was consistent with that of the extracapsular fat. Distinction between stage I and II tumors be evaluated by comparing the energy spectrum curves of the mass and the extracapsular fat of the mass. The accuracy rate of is 79.4%. For stages III and IV, there was no significant difference in the slope of the energy spectrum curve of the tumor parenchyma, metastatic lymph node, and intravascular embolism (P>0.05). The energy spectrum curve of the tumor parenchyma was consistent with that of the enlarged lymph nodes and intravascular emboli. The two radiologists have strong consistency in evaluating TETs Masaoka-Koga staging, The Kappa coefficient is 0.873,(95%CI:0.768-0.978).

**Conclusion:**

Spectral CT parameters, especially the energy spectrum curve and slope, are valuable for preoperative TET and can be used in preoperative staging prediction.

## Introduction

Thymic epithelial tumors (TETs), which include thymoma and thymic cancer, originate from thymic epithelial cells and are composed of different proportions of epithelial cells and lymphocytes. TETs are the most common tumors in the anterior mediastinum, accounting for 47% of tumors in this location (1). However, their overall incidence is not high, accounting for 1.3–2.2/10^6^ cases (2, 3). TETs are common in elderly people but rare in children and have been reported in patients aged 10 to 80 years, with an average age of 50 to 60 years among men and women (4). Although some patients present with paraneoplastic autoimmune diseases, most patients have no clinical symptoms. Among these diseases, myasthenia gravis is the most common, affecting approximately one-third of patients. Surgery remains the most common treatment for TETs. Thoroughness of tumor resection is an important factor that influences the prognosis of patients, as the prognosis is significantly better after complete resection than after incomplete resection (5). Tumor diameter, tumor resection style, World Health Organization (WHO) classification, Masaoka-Koga stage, and postoperative radiotherapy and chemotherapy are independent factors that affect the prognosis of patients with thymoma. As a result of the correlation between the CT imaging characteristics of TETs and clinical stage (6, 7), tumor stage is evaluated before surgery based on imaging parameters and appropriate treatment strategies are applied. Computed tomography (CT) is currently the preferred method for identifying and characterizing thymic tumors and provides higher sensitivity and specificity (8). The current CT-based staging approach for TETs is based on the appearance of tumor images, and they remain difficult to accurately stage; magnetic resonance imaging (MRI) combined with diffusion-weighted imaging and apparent diffusion coefficient values can also be used in quantitative analyses (9). The staging of TETs using MRI is more accurate than that when using CT; however, MRI scanning requires higher, for example, longer inspection time and restrictions on metal foreign bodies in the body. More convenient, non-invasive, and quantifiable indicators are necessary for preoperative staging.

There are many staging systems for TETs (10), and the most commonly used is the Masaoka-Koga staging system. The Masaoka-Koga staging system is based on the tumor invasion range, surrounding structure invasion and implantation, and distant metastasis, among other factors (8, 11, 12). The latest staging system is the eighth edition of the TNM staging system (13). A recent study (14) collected 217 responses from 37 countries in four continents; 78% of scholars thought the TNM classification was useful (N=169), and 87% of scholars still use the Masaoka-Koga staging system (N=189). Staging is related to the prognosis of TETs (9, 15), and the treatment of different stages of disease are also different. Surgery is generally considered an effective treatment method (16, 17); however, some researchers have pointed out that for some patients with thymoma, surgical treatment may not be the best treatment method (18). It has been reported that for patients with stage I thymoma according to the Masaoka-Koga staging, open surgical resection carries the risk of potential surgical damage and other complications, and CT-guided percutaneous radiofrequency treatment of stage I thymoma is related to minor trauma, fewer complications, and good treatment results (19). In patients with Masaoka-Koga stage I and II thymoma, after appropriate surgical resection, auxiliary radiation treatment has no obvious effect on outcomes (20). Platinum-based chemotherapy remains the standard first-line treatment for patients with advanced or metastatic TETs (21).

Based on the correlation between stage and tumor treatment decisions and prognosis, it is necessary to use non-invasive imaging methods to predict the stage before surgery; CT is currently the preferred method for identifying and characterizing thymic tumors, providing higher sensitivity and specificity (8, 22). Preoperative CT analysis and understanding of the tumor margin, capsule, surrounding invasion levels, pleural implants, lymph nodes, and distant metastases can predict the stage of TETs and have a positive effect on patient treatment and prognosis (23). However, conventional CT is subjective and is based on characterization of the lesion, multiple spectral CT variables can be used to quantify tumor indicators and more accurate preoperative staging.

## Materials and Methods

This study was approved by the institutional review board, and the requirement for written informed consent was waived due to the retrospective nature of this study.

### General Information

Sixty-two patients with surgically and pathologically confirmed TETs treated in our hospital from October 1, 2014 to December 31, 2020 were enrolled; among these, eight patients with insufficient image quality for regions of interest (ROIs) to be drawn were excluded. Finally, 54 patients were included, comprising 27 men and 27 women aged from 37 to 73 years old, with an average age of 55.56 ± 9.79 years. According to the Masaoka-Koga staging system, 19 patients had stage I, 15 patients had stage II, 8 patients had stage III, and 12 patients had stage IV disease. The 54 patients were divided into three groups: non-invasive group (stage I), surrounding fat group (stage II), and surrounding structures and distant metastasis group (stages III and IV). The most common clinical manifestations were intermittent cough, chest tightness, chest pain, and shortness of breath. Eight patients had myasthenia gravis, which manifested as drooping eyelids, limb weakness, and difficulty chewing. Seven of these patients were identified during physical examination, and three patients had recurrence. All patients were treated surgically, and the specimens were sent for pathological examination postoperatively.

### Instruments and Methods

All patients underwent plain and dual-phase-enhanced energy spectrum CT (Discovery CT 750 HD; GE Healthcare, Waukesha, WI, USA). Patients were supine during the scan, which was conducted from the apex to the bottom of the lung. The scanning parameters were as follows: flat scan tube at a voltage of 120 kV, enhanced scan tube at a voltage of 80/140 kV with fast switching, rack speed of 0.6 s/r, tube current of 325 mA, pitch of 0.983:1, detector coverage of 40 mm, collimator width of 1.25 mm, and reconstruction layer thickness and layer spacing of 1.25 mm. An iodinated contrast agent (Ultravist 370, Bayer Schering Pharma, Berlin, Germany) was used at a flow rate of 3–4 mL/s and a dose of 1.0 mL/kg injected *via* the anterior cubital vein using a high-pressure syringe (Ulrich Medical, Ulm, Germany). The trigger threshold for thoracic aorta monitoring was 80 Hounsfield units. The arterial phase scan was performed 8 s after the trigger, while the venous phase scan was performed 30 s after the trigger.

### CT Image Analysis

Two radiologists used the AW 4.6 workstation GSI Viewer software to perform CT scans. ROIs 5×5 mm in size were delineated in homogeneous lesions while avoiding necrotic cysts, calcification, and vascular shadows. ROIs in the thoracic aorta, the fat layer within 10 mm outside the lesion capsule, and the fat layer on the anterior chest wall were delineated with 5×5-mm ROIs. Enlarged lymph nodes and intravascular emboli were outlined by ROIs of the same size. Each ROI was drawn three times to obtain an average value. The single-energy CT value, energy spectrum curve, and iodine concentration in the ROI was recorded. The standardized iodine concentration (NIC) was determined using the IC _lesion_/IC _chest_ equation, where IC _lesion_ is the iodine concentration in the lesion and IC _chest_ is the iodine concentration in the thoracic aorta. The slope was calculated as (CT_40_−CT_100_)/(40−100), where CT_40_ is the CT value at 40 keV and CT_100_ is the CT value at 100 keV.

### Pathological Images

Pathological images were first observed under low and high magnification by a pathologist with 3–5 years of work experience. Diagnosis was based on the WHO histological classification. Microscopic observation was performed to assess whether the tumor had invaded the surrounding fat and connective tissue, and whether it had invaded the mediastinal pleura, pericardium, and lungs. It was also clarified whether an intravascular embolus was a thrombus or a tumor, and whether there was metastasis to the lymph nodes. The diagnosis was confirmed by a pathologist with more than 10 years of work experience.

### Masaoka-Koga Stage

In combination with the postoperative pathology, tumor staging was performed based on whether the tumor capsule was intact during surgery, the surrounding fatty layer was clear, and the mediastinal pleura, pericardium, lungs, and blood vessels were adherent and extensively invaded (8, 11, 12). Stage I tumors were defined as those where the capsule was intact and no extracapsular invasion was observed under the microscope. Stage II tumors were defined as those where macroscopic and microscopic invasion of the mediastinal adipose tissue could be observed, with no invasion of the mediastinal pleura or pericardium. Stage III tumors were defined as those with visible invasion of adjacent structures (such as the pericardium, large blood vessels, or lungs) were observed. Stage IV-A tumors were defined as those with pleural dissemination (pleural or pericardial metastasis) (was observed) and stage IV-B tumors were defined as those with lymph or blood-based metastasis to a location outside the thoracic region was observed. Based on the extent of tumor invasion, the tumors were divided into a non-invasive group (stage I), invasion of the surrounding fat group (stage II), and invasion of surrounding structures (pleura, pericardium, lung, blood vessels) and distant metastasis group (stages III and IV).We performed TNM staging based on all data and performed a consistency analysis with Masaoka-Koga staging.

### Statistical Methods

SPSS 23.0 software was used for analysis; t-test was used for quantitative comparisons between two groups. Multiple sets of quantitative data were analyzed by one-way analysis of variance, and variances were analyzed using the Kruskal-Wallis test. Pairwise comparisons between groups were performed using the Bonferroni method. The Kappa value was used to evaluate the agreement between two observers. Calculate the accuracy of the energy spectrum CT parameters for TETs staging. P < 0.05 indicating that the differences were statistically significant.

## Results

For all 54 cases, there was a strong consistency between TNM staging and Masaoka-Koga staging (ICC=0.852). According to TNM staging, there were more stage I cases than according to the Masaoka-Koga staging. In the two staging methods, there was no significant change in the data for stages III and IV. The two radiologists have strong consistency in evaluating TETs Masaoka-Koga staging, The Kappa coefficient is 0.873 (95% CI: 0.768-0.978).

### Energy Spectrum CT Results for Three Groups of Lesions

The single-energy CT values at 40-140 keV, NIC, and slope of the energy curve in the non-invasive group (stage I), surrounding fat group (stage II), and surrounding structures (pleura, pericardium, lung, blood vessels) and distant metastases group (stages III and IV) were not significantly different (**Table 1**).

**Table 1:** Spectral CT multi-parameter results of the three groups in stages (Arterial phase).

Parameters	Non-invasive group (stage I)	Invasion of the surrounding fat group (stage II)	Invasion of surrounding structures and distant metastasis groups (stage III和IV)	F/H	P
40 keV	179.51 ± 48.56	177.28 ± 43.52	169.24 ± 39.12	0.291	0.749
50 keV	130.22 ± 31.71	129.98 ± 28.93	123.04 ± 25.68	0.373	0.691
60 keV	98.86 ± 21.42	99.53 ± 21.25	92.15 ± 18.06	0.744	0.481
70 keV	79.87 ± 16.32	80.90 ± 15.92	74.10 ± 13.39	1.044	0.360
80 keV	68.60 ± 14.05	70.30 ± 12.96	64.67 ± 10.92	0.892	0.416
90 keV	61.11 ± 12.61	63.63 ± 11.55	58.23 ± 10.35	0.889	0.418
100 keV	55.56 ± 12.00	58.28 ± 10.84	53.06 ± 9.81	0.912	0.408
110 keV	51.65 ± 11.76	54.49 ± 10.44	49.38 ± 9.59	0.915	0.408
120 keV	48.89 ± 11.71	51.88 ± 10.23	46.80 ± 9.51	0.916	0.407
130 keV	46.77 ± 11.72	49.85 ± 10.15	44.81 ± 9.48	0.906	0.411
140 keV	45.13 ± 11.75	48.26 ± 10.07	43.27 ± 9.49	0.891	0.417
NIC	0.15 ± 0.05	0.12 ± 0.04	0.13 ± 0.04	1.191	0.313
Curve slope	-2.06 ± 0.74	-1.98 ± 0.63	-1.93 ± 0.58	0.191	0.827

### CT Results for Stage I and Stage II Lesions, Lesion Extracapsular Fat, and Anterior Chest Wall Fat

**Figure 1:**
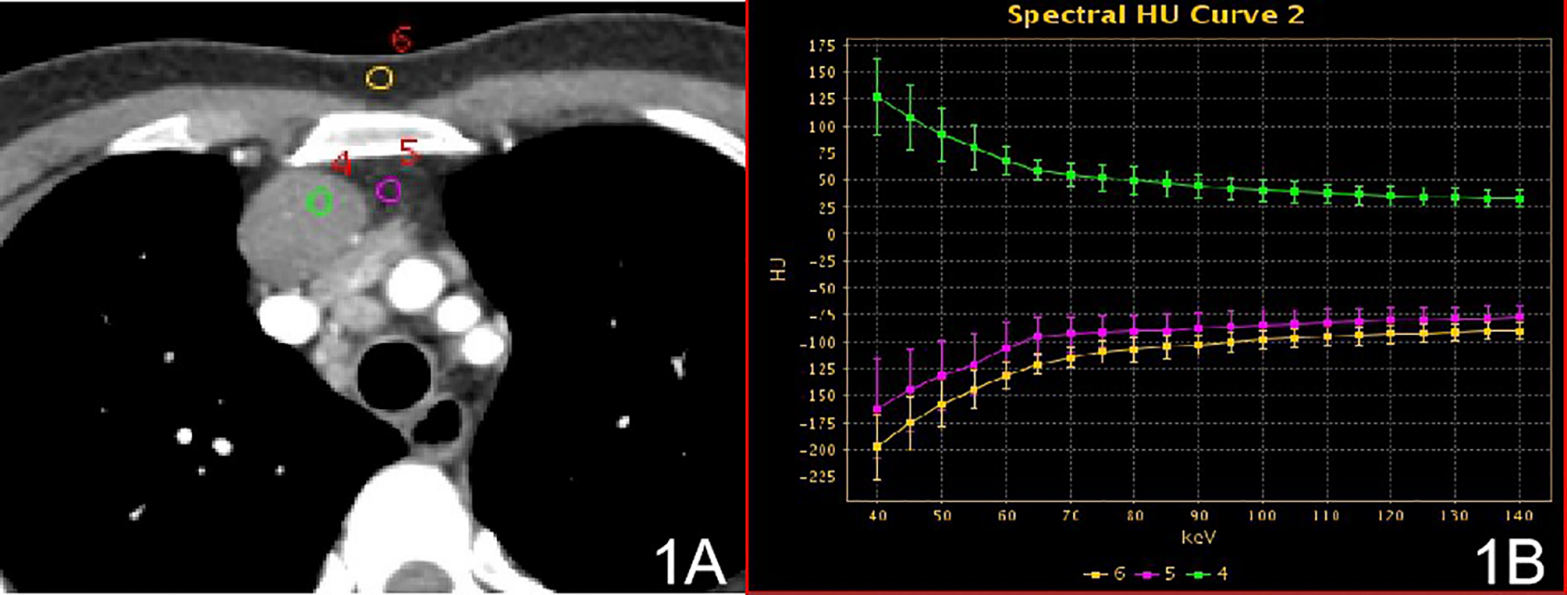
Stage I, **(A)** 70 keV single-energy CT value of the arterial phase is used to outline the ROI of the lesion; **(B)** spectrum of arterial lesions (green), spectrum of fat outside the lesion (purple) and spectrum of anterior chest wall fat (yellow).

**Figure 2:**
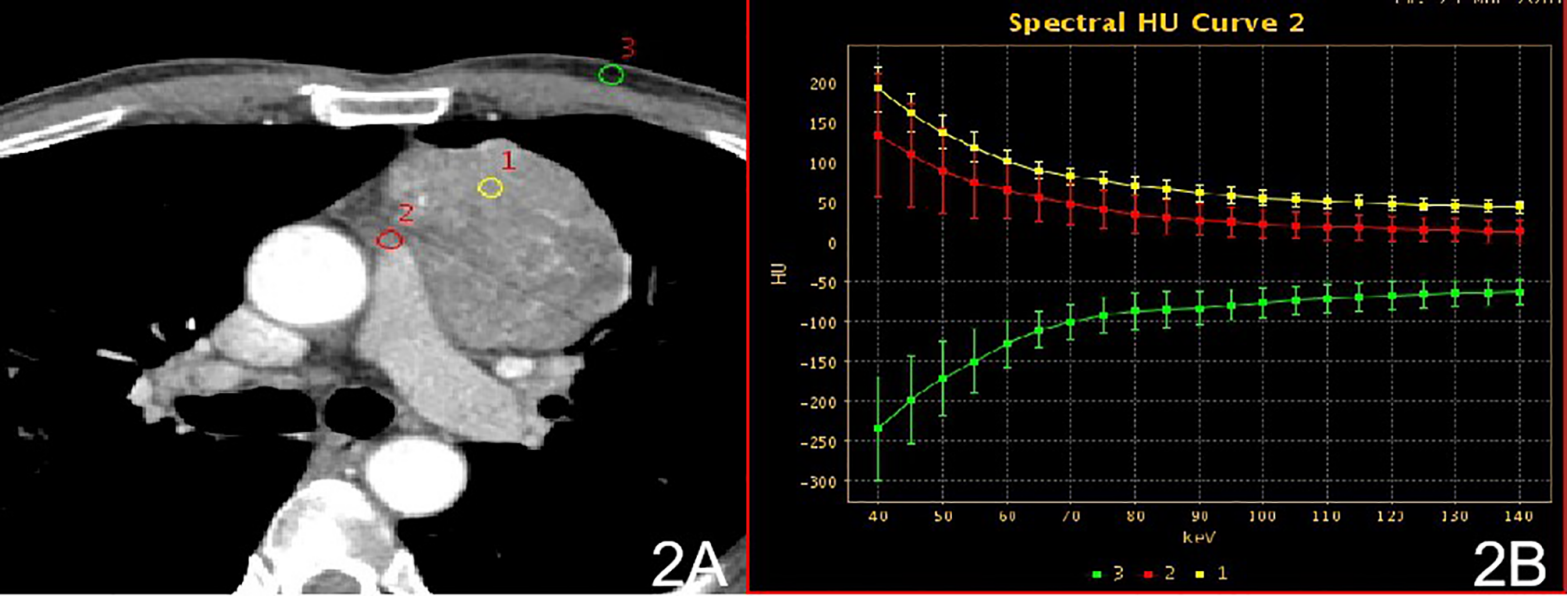
Stage II, **(A)** 70 keV single-energy CT value of the arterial phase is used to outline the ROI of the lesion; **(B)** arterial lesion spectrum (yellow), extra-focal fat spectrum (red) and anterior chest wall fat spectrum (green).

In stage I, the difference in the slope of the energy spectrum curve of tumor parenchyma, tumor extracapsular fat, and anterior chest wall fat was statistically significant (P < 0.001, P < 0.001) (**Table 2**) **(****Figure 1**). In stage II, the difference between the tumor parenchyma, the fat outside the tumor, and the slope of the spectrum curve of the anterior chest wall was statistically significant (P < 0.001, P < 0.001) (**Table 3**) (**Figure 2**). According to the slope of the energy spectrum curve of the outer fat layer of the lesion, combined with the energy spectrum curve graph, the accuracy of spectral CT for predicting the Masaoka-Koga stage I and II lesions was 79.4%.

**Table 2:** Slopes of energy spectrum curves, extracapsular fat and anterior chest wall fat of TETs stage I.

	Slope of the energy spectrum curve of the lesion	Slope of energy spectrum curve of extracapsular fat in lesions	Slope of energy spectrum curve of anterior chest wall fat	F/H	*P*
Arterial phase	-2.00 ± 0.76	1.94 ± 0.69*	2.32 ± 0.33	293.52	0.000
Venous phase	-2.10 ± 0.51	1.72 ± 0.48*	2.08 ± 0.33	500.89	0.000

Compared with the slope of the lesion energy spectrum curve, *P < 0.05; Compared with the slope of the anterior chest wall energy spectrum curve, ^#^P < 0.05.

**Table 3:** Slopes of energy spectrum curves of, extracapsular fat and anterior chest wall fat TETs stage II.

	Slope of the energy spectrum curve of the lesion	Slope of energy spectrum curve of extracapsular fat in lesions	Slope of energy spectrum curve of anterior chest wall fat	F/H	P
Arterial phase	-1.91 ± 0.66	-1.27 ± 0.57*	2.18 ± 0.27	223.54	0.000
Venous phase	-2.27 ± 0.48	-1.46 ± 0.53*#	2.05 ± 0.28	409.71	0.000

Compared with the slope of the lesion energy spectrum curve, *P < 0.05; Compared with the slope of the anterior chest wall energy spectrum curve, ^#^P < 0.05.

### CT Results for Stage IV Lesions, Intravascular Tumor Thrombi, and Metastatic Lymph Nodes

**Figure 3:**
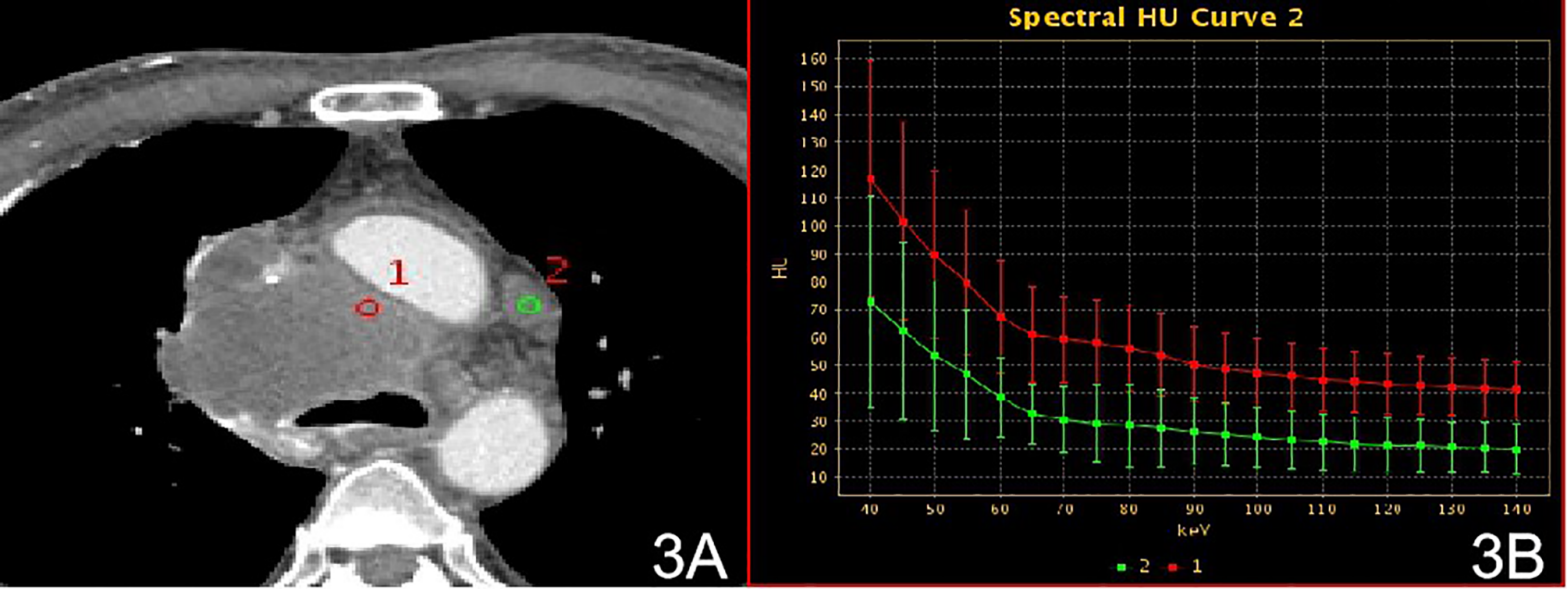
Stage IV, **(A)** 70 keV single-energy CT value of the arterial phase is used to outline the ROI of the lesion; **(B)** arterial lesion spectrum (red), lymph node spectrum (green).

**Figure 4:**
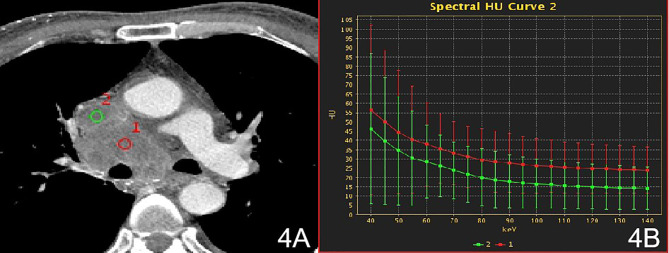
Stage IV, **(A)** 70 keV single-energy CT value of the arterial phase is used to outline the ROI of the lesion; **(B)** Arterial lesion spectrum (red), intravascular emboli spectrum (green).

There were no significant differences in the slopes of the energy spectrum curves for stage IV lesions, metastatic lymph nodes, and intravascular emboli (P > 0.05) (**Table 4**) (**Figures 3** and **4**).The slope of the energy spectrum curve combined with the energy spectrum curve graph, the accuracy of assessing lymph node metastasis and embolic properties is 80.0%.

**Table 4:** Slope analysis of spectrum curves of lesions, metastatic lymph nodes, and intravascular emboli in group 3 (stages III and IV).

	Slope of the energy spectrum curve of the lesion	Slope of the energy spectrum curve of metastatic lymph nodes	Slope of the energy spectrum curve of intravascular emboli	F/H	P
Arterial phase	-1.83 ± 0.64	-1.53 ± 0.68	-1.45 ± 0.65	0.964	0.393
Venous phase	-1.72 ± 0.59	-1.69 ± 0.57	-1.37 ± 0.77	2.482	0.101

## Discussion

Three parameters were evaluated in this study, the single energy CT value of the lesion, the NIC, and the slope of the energy spectrum curve. The single-energy imaging mode of energy spectrum CT simulates the image of an object in the presence of a monochromatic X-ray source. The attenuation of different tissues changes in accordance with changes in the X-ray beam energy. A lower single-energy beam can increase the density resolution of the image, which helps to show lesions. The slope of the energy spectrum curve reflects the magnitude of the enhancement. The larger the slope is, the greater the magnitude of the enhancement will be, and the energy spectrum curve of the tissue changes greatly at low energy. Therefore, low energy (40-100 keV) was selected to determine the slope of the energy spectrum curve for each group. The shape-like energy spectrum curve may reflect the homology of the region of interest. The iodine (water) concentration of the lesion was directly obtained from the material separation map for the energy spectrum CT, which quantitatively reflects the iodine uptake and distribution in the lesion. To eliminate individual differences, the NIC was used to compare the iodine (water) base values. The NIC was more reliable than the iodine concentration (24). NIC is an indirect reflection of the iodine content of a lesion, that is, the degree of strengthening.

The 54 patients evaluated in this study included 19 with stage I, 15 with stage II, 8 with stage III lesions, and 12 with stage IV lesions according to the Masaoka-Koga staging. The 54 patients were divided into three groups: non-invasive group (stage I), invasive surrounding fat group (stage II), and invasive surrounding structures (pleura, pericardium, lung, blood vessels) and distant metastasis group (stages III and IV). In the qualitative diagnosis, irregular or lobed edges lesions on conventional CT. It may indicate that the tumor capsule and extracapsular fat have been invaded (25). In this study, ROIs were delineated in stage I and stage II solid lesions, extracapsular fat, and anterior chest wall fat. The energy spectrum curves for the lesion, extracapsular fat, and anterior chest wall fat were obtained. In stage I lesions, the energy spectrum curve of the tumor parenchyma and the energy spectrum curve of the extracapsular fat have the opposite shape, and the energy spectrum curve of the extracapsular fat and the front chest wall fat have the same shape, indicating that the lesion did not extend outside the capsule. The slope of the spectrum curve for the anterior chest wall fat in stage II lesions showed statistically significant differences with those of the spectra for the lesion and the lesion’s extracapsular fat, indicating that the lesion had invaded the extracapsular fat. According to the spectrum curve, the lesion and the fat in the outer capsule of the lesion were homologous. In this study, ROIs were delineated in pathologically confirmed mediastinal lymph node with metastasis, intravascular tumor emboli, and solid lesions. The spectral curves for the metastatic lymph nodes, intravascular emboli, and lesions were consistent and the differences in the slopes were not statistically significant, indicating that the lesions were homologous to lymph nodes and intravascular emboli (26). Among these parameters, the energy spectrum curve objectively reflects whether the tumor parenchyma and extracapsular fat are homologous, and whether the enlarged lymph nodes and emboli are metastatic.

In conclusion, distinction between stage I and II tumors may be evaluated by comparing the energy spectrum curves of the mass and the extracapsular fat of the mass. Areas of interest should be outlined in suspicious enlarged lymph nodes and intravascular emboli before surgery to determine whether the lymph nodes have metastasized and the nature of the emboli in the vessel.

### Limitations

The limitations of our study are as follows: This study was a retrospective and single-center study, lacking multi-center data verification and the sample size was small. The accuracy of preoperative predicting staging needs further large sample size research. Measurement of the ROI of fat outside the capsule stays a difficult points, even though we used the average value of 3 ROIs of the same size drawn in the fat layer 10 mm outside the capsule of the lesion to reflect as much as possible whether the lesion has extracapsular fat invasion.

## Data Availability Statement

All data generated or analyzed during this study are included in this published article.

## Author Contributions

QZ designed the study, performed the literature search and data analysis, and drafted the manuscript. JZ and QZ participated in the conception and design of the study, performed the quality assessment and helped to revise the manuscript. JM, FW, BZ and XK performed the study selection and statistical analysis and helped to draft the manuscript. QZ, JM, XK, BZ, and FW performed the data extraction and analysis, modified the language. All authors contributed to the article and approved the submitted version.

## Funding

This work was supported the National Natural Science Foundations of China(81772006).

## Conflict of Interest

The authors declare that the research was conducted in the absence of any commercial or financial relationships that could be construed as a potential conflict of interest.
